# COVID-19 and Death Anxiety: The Impact on Students’ Appraisals to Completing Advance Directives

**DOI:** 10.1093/geroni/igab046.514

**Published:** 2021-12-17

**Authors:** Carlyn Vogel, Debra Dobbs, Maureen Templeman, Victoria Marino, William Haley

**Affiliations:** 1 University of South Florida, Tampa, Florida, United States; 2 University of South Florida, School of Aging Studies, University of South Florida, Florida, United States

## Abstract

This study examined possible effects of COVID-19 on students’ appraisals, coping, and responses to completing advance directives (ADs). We used the transactional model of stress and coping to explore 93 undergraduate students’ responses to an AD assignment completed in an undergraduate course during COVID-19. Students watched a recorded lecture, read content related to ADs, and examined a sample copy of a 5 Wishes document. Students completed an assignment reflecting on reactions to completing ADs. Content analysis of 65 responses indicated almost 10% of students mentioned COVID-19 or the pandemic as a reason to complete ADs. Approximately 18% mentioned their youth and 40% mentioned sudden or serious illness as reasons to complete ADs. Nearly 30% mentioned death anxiety as a reason for being unprepared to complete ADs. Instructors should consider ways to inform and help students process their emotions given contextual factors (e.g. the pandemic) when teaching about ADs.

